# Imbalanced Seismic Event Discrimination Using Supervised Machine Learning

**DOI:** 10.3390/s22062219

**Published:** 2022-03-13

**Authors:** Hyeongki Ahn, Sangkyeum Kim, Kyunghyun Lee, Ahyeong Choi, Kwanho You

**Affiliations:** 1 Department of Electrical Computer Engineering, Sungkyunkwan University, Suwon 16419, Korea; ahk5721@skku.edu (H.A.); interpost94@skku.edu (S.K.); naman2001@skku.edu (K.L.); overcld7@skku.edu (A.C.); 2 Department of Smart Fab. Technology, Sungkyunkwan University, Suwon 16419, Korea

**Keywords:** seismic discrimination, artificial explosion, oversampling method, supervised machine learning

## Abstract

The discrimination between earthquakes and artificial explosions is a significant issue in seismic analysis to efficiently prevent and respond to seismic events. However, the discrimination of seismic events is challenging due to the low incidence rate. Moreover, the similarity between earthquakes and artificial explosions with a local magnitude derives a nonlinear data distribution. To improve the discrimination accuracy, this paper proposes machine-learning-based seismic discrimination methods—support vector machine, naive Bayes, and logistic regression. Furthermore, to overcome the nonlinear separation problem, the kernel functions and regularized logistic regression are applied to design seismic classifiers. To efficiently design the classifier, P- and S-wave amplitude ratios on the time domain and spectral ratios on the frequency domain, which is converted by fast Fourier transform and short-time Fourier transform are selected as feature vectors. Furthermore, an adaptive synthetic sampling algorithm is adopted to enhance the classifier performance against the seismic data imbalance issue caused by the non-equivalent number of occurrences. The comparisons among classifiers are evaluated by the binary classification performance analysis methods.

## 1. Introduction

Seismic signal analysis is one of the significant problems in geology. In particular, several studies have been conducted to discriminate between earthquakes and artificial explosions, and the results have exhibited the importance of seismic discrimination on the grounds of preparation and damage minimization caused by an earthquake. As earthquakes show aperiodic and non-stationary characteristics, the work of seismic event classification should be immediately performed. One factor that interferes with the classification is an artificial explosion. Artificial explosions, e.g., quarry blasts, explosive bomb tests, and underground nuclear tests, accompany seismic tremors, and the surface oscillations are similar to earthquakes [1].

Due to the analogous characteristics between earthquakes and artificial explosions, seismic stations that record and analyze various seismic waves can misidentify seismic events. Therefore, seismic signal discrimination needs to be performed before the precision analysis and initial response to the event. Typical seismic discrimination is performed by visually analyzing the recorded signals of earthquakes and explosions or extracting the characteristics of each record [2].

The process consumes a substantial amount of time and requires a large number of seismic data. To reduce the analysis time and to discriminate precisely between earthquakes and artificial explosions, state-of-the-art machine-learning methods have been applied to design classifiers from seismic datasets. Many machine-learning-based discrimination methods have been introduced to discriminate between earthquakes and artificial explosions [3,4,5,6].

Li [3] conducted a classifier to discriminate earthquakes and several noises using a generative adversarial network for early warning system. Lyubushin [4] proposed a linear Bayesian discriminator. The discriminator was based on the properties of multi-fractal singularity spectrums that represent the fractal dimension of time moments. Lindenbaum [5] suggested a neural-net-based deep canonical correlation for automatic discrimination. A signal-to-noise ratio estimator and a short-time average/long-time average detector were introduced to identify triggers for seismic events. Bergman [6] induced an array-based seismic discrimination method using diffusion maps to discriminate between earthquakes and explosions. To handle the diffusion map, this work included a pre-processing step in which P-wave and S-wave analysis was done under a time-frequency representation.

However, seismic events do not occur evenly. In partciular, the incidence rate for artificial explosions becomes lower than for earthquakes in regions where the earth’s interior and plate tectonics are activated. Conversely, artificial explosions often occur more frequently than earthquakes in certain regions [7]. Furthermore, the small-scale seismic events that are measured under local magnitudes ($m_{l}$) with less than 3.0 make it difficult to analyze features linearly. Therefore, a significant incidence discrepancy implies that data imbalances occur between earthquakes and artificial explosions.

The imbalance problem results in the overfitting or underfitting problem. Therefore, the discrimination accuracy of seismic signals decreases [8]. In this paper, support vector machine (SVM), naïve Bayes (NB), and logistic regression (LR) are used as seismic classifiers to discriminate binary classification.

In addition, SVM, NB, and LR-based discrimination methods are executed using limited seismic data recorded from a single station as a training dataset for the rapid response. Furthermore, to overcome the nonlinear data distribution caused by the similarity between earthquake and artificial explosion signals, the regularized logistic regression (RLR) and kernel methods for SVM are applied to construct seismic discrimination system. For supervised machine-learning methods to improve the seismic discrimination accuracy, the feature vector is obtained from the time- and frequency-domain-based amplitude ratio.

Furthermore, to prevent errors caused by domain change between time and frequency, fast Fourier transform (FFT) and short-time Fourier transform (STFT) are applied to reduce the conversion time and visualize the frequency band of the seismic signal, respectively. However, earthquakes and artificial explosions exhibit different seismic stroke incidence rates. Differences in the incidence rate lead to the imbalances in the dataset that can bring unintended error in designing a machine-learning-based seismic classifier.

To handle the imbalanced dataset, in this paper, the adaptive synthetic sampling (ADASYN) algorithm is applied to machine-learning algorithms in order to reduce the misclassification. To maintain a balance between majority class and minority class, the synthetic dataset is generated from the minority class using the ADASYN algorithm. The supervised training techniques derived in this paper are compared and evaluated to identify an optimal machine-learning-based seismic classifier between earthquakes and artificial explosions.

To determine the optimal machine-learning method for imbalanced seismic dataset, this paper is organized as follows. Section 2 analyzes the seismic signal in the time and frequency domain to form feature vector of machine-learning classifiers. Section 3 presents seismic discrimination methods using four different ADASYN-based machine-learning algorithms. The results of machine-learning classifiers are compared using evaluation indexes. Furthermore, the ADASYN algorithm demonstrates the solution of the imbalanced dataset problem and increases classification performance in Section 4. Finally, our conclusions are presented in Section 5.

## 2. Seismic Signal Discrimination

A seismic wave refers to the energy flow through the surface. Seismic events occur due to various causes, such as earthquakes caused by natural phenomena and artificial explosions caused by man-made situations. Although the causes of seismic events are different, the signals of seismic waves exhibit similar characteristics.

In seismology, several approaches have been attempted to demonstrate the difference between earthquakes and artificial explosions to determine the inherent characteristics of each seismic event [9,10,11]. To distinguish between earthquakes and artificial explosions, the amplitude ratio ($A_{r}$) and spectral ratio ($S_{r}$) for P- and S-waves that represent seismic characteristics are used to derive seismic type.

**Figure 1:**
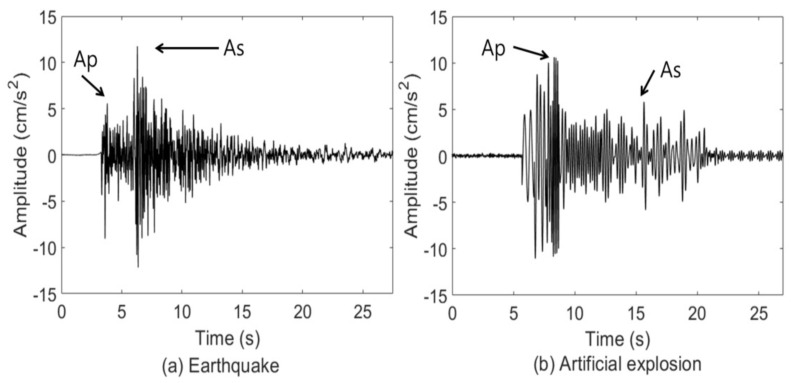
Peak amplitudes of P- and S-waves.

Many studies have shown that earthquakes and artificial explosions have distinct peaks in amplitude. The $A_{r}$ method in [12,13] is based on the amplitude of P-wave and S-wave in the seismic signal in the time domain. The earthquake with a specific magnitude band (1.8<m<3.0) and local distance from 50 km to 200 km, in general, exhibits a peak amplitude of P-wave ($A_{p}$) less than the peak amplitude of S-wave ($A_{s}$). In contrast, the artificial explosion has a larger $A_{p}$ than $A_{s}$. Based on the distinct peak values, the $A_{r}$ method is used to obtain the peak amplitude ratio. Figure 1 shows the different peak amplitudes of earthquakes and artificial explosions, respectively.

The $S_{r}$ method is similar to the $A_{r}$ method except that it is based on the amplitude ratio of the seismic signal in the frequency domain. For the general seismic events, the energy of S-wave, which has a low frequency compared to the P-wave, is higher than the energy of the P-wave. In addition, the duration of an earthquake is longer than an artificial explosion [14]. According to the duration and energy difference, the $S_{r}$ of the artificial explosion is more affected by the P-wave as compared to the earthquake. Based on the amplitude ratio in the frequency domain, the $S_{r}$ method in Equation (1) is designed as the following frequency bands.

(1) $$S_{r}=\frac{\int_{f_{h1}}^{f_{h2}}Amp\left(f\right)df}{\int_{f_{l1}}^{f_{l2}}Amp\left(f\right)df},$$

where Amp(f) represents the amplitude of the frequency domain and $f_{h1},f_{h2},f_{l1}$ and $f_{l2}$ denote the high-frequency and low-frequency bands. In general, the Fourier transform (FT) is used to represent the signal in the frequency domain. However, the time-frequency data is difficult to obtain from FT, since the FT is a function of frequency *f* and does not include the continuity property over time. Furthermore, when the signal data contain discontinuity and high-frequency components, the analysis of the signal’s features becomes more complicated.

The FFT algorithm is adopted to prevent data loss caused by the conversion of the domain and overcome the weakness of the FT. In addition, STFT algorithm is applied to the seismic signal to acquire the optimized frequency band in the seismic signal. The *STFT* of the seismic signal f(t) is expressed as follows [15]:

(2) $$STFT\left\{f(t)\right\}\left(\tau ,f\right)=\int_{-\infty }^{\infty }f\left(t\right)\rho \left(t-\tau \right)e^{-j2\pi ft}dt,$$

where ρ(t) represents a window function of the analysis. The resolution of the STFT is affected by the shape and size of the window function. When the STFT is applied, the transformed data are represented as a spectrum in the time-frequency division since the STFT is based on the FT in each time interval. Therefore, we can show the frequency change of the given data as a frequency spectrum. Based on the frequency spectrum, the optimized frequency bands are determined to obtain the $S_{r}$ data of seismic signals.

## 3. Implementation of ADASYN-Based Classification Algorithms

**Figure 2:**
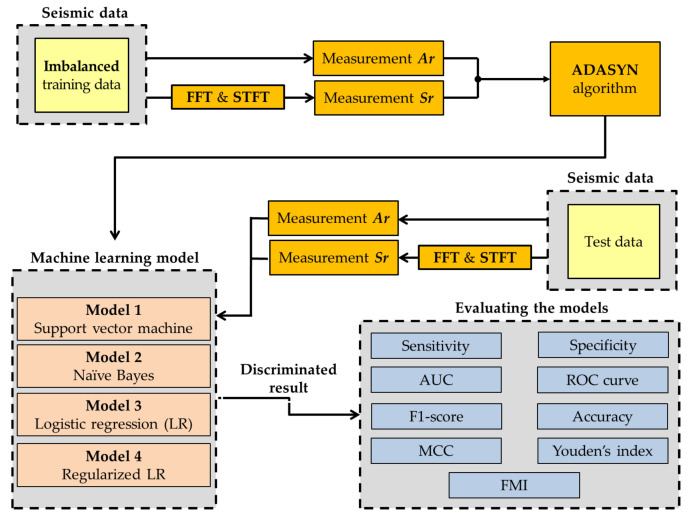
A flowchart of the proposed seismic discrimination.

To implement the machine-learning algorithms to distinguish between earthquake as the majority class and artificial explosions as minority class, four separate machine-learning algorithms are adopted as illustrated in Figure 2. As a first step, an imbalanced seismic dataset is collected as a training dataset. To determine out the feature vectors, the $A_{r}$ method in the time domain and the $S_{r}$ method in the frequency domain converted using FFT and STFT are applied, respectively. The training dataset can be expressed as,

(3) $$D=\left[\begin{array}{cc} d_{11} & d_{12}\\ d_{21} & d_{22}\\ \vdots & \vdots \\ d_{n1} & d_{n2} \end{array}\right]=\left[\begin{array}{c} d_{1}\\ d_{2}\\ \vdots \\ d_{n} \end{array}\right],\;\;l=\left[\begin{array}{c} l_{1}\\ l_{2}\\ \vdots \\ l_{n} \end{array}\right],\;\;l_{i}\in \left\{+1,-1\right\},$$

where the element ($d_{i}$) of *D* is a two-dimensional feature vector that describes $A_{r}$ and $S_{r}$ value. $l_{i}$ is a label with +1 that denotes an earthquake and −1 that represents an artificial explosion for i∈{1,2,⋯, *n*}. *n* denotes the number of training data. Second, the ADASYN algorithm is used to generate the synthetic dataset on minority class for the balance between majority class and minority class.

As a third step, classifiers based on SVM, NB, LR, and RLR with ADASYN are applied. Finally, a new seismic dataset that includes $A_{r}$ and $S_{r}$ is used to evaluate the performance of the model. For the evaluation of the classification model, nine methods, such as the specificity, sensitivity, receiver operating characteristic (ROC) curve, area under the curve (AUC), F1-score, accuracy, Matthews correlation coefficient (MCC), Youden’s index (YI), and Fowlkes–Mallows index (FMI), are used as performance indicators of binary classification and a comparison value of each seismic discrimination performance.

### 3.1. Support Vector Machine

SVM is a supervised and non-probabilistic classification model for the machine learning. Several studies have used SVM to solve the classification problem and distinguish the dataset as true or false in various fields [16].

To compose the classifier using an SVM, a hyperplane in the feature space between two classes of data is determined by a weighting vector ω. The weighting vector is derived with the maximum margin between the support vector and hyperplane to minimize the measurement uncertainty caused by the noise components in the measured dataset. The hyperplane is written as $\omega \cdot d_{i}+b=0$, where *b* is the unregulated bias term. The separating hyperplane can be defined as two classes, which are $l_{i}=+1$ and $l_{i}=-1$, respectively,

(4) $$l_{i}=\left\{\begin{array}{cc} +1, & \;\mathrm{for}\;\;\omega d_{i}+b\geq 1,\\ -1, & \mathrm{for}\;\;\omega d_{i}+b\leq -1. \end{array}\right.$$

$l_{i}\left(\omega \cdot d_{i}+b\right)-1\geq 0$ is derived by combining the inequality condition in Equation (4). The margin $2/∥\omega ∥$ between the hyperplanes needs to be maximized to solve the constrained optimization problem under inequality constraints. To derive the maximum margin, the constrained optimization problem can be written as

(5) $$\begin{array}{ccc} \mathrm{maximize} & \; & \frac{2}{∥\omega ∥},\\ \mathrm{subject}\;\mathrm{to} & \; & l_{i}\left(\omega \cdot d_{i}+b\right)\geq 1. \end{array}$$

Lagrange multipliers in the Karush–Kuhn–Tucker (KKT) condition are applied to maximize the objective function. Equation (5) can be transformed into ${∥\omega ∥}^{2}/2$, which represents the converted form of the minimization, and the Lagrange function is

(6) $$L\left(\omega ,b,\alpha \right)=\frac{{∥\omega ∥}^{2}}{2}-\sum_{i=1}^{n}\alpha_{i}\left[l_{i}\left(\omega \cdot d_{i}+b\right)-1\right],$$

where $\alpha_{i}$ is the Lagrange multiplier, that is nonzero for support vectors [17]. According to the complementary slackness and stationarity in the KKT condition and dual solution, the decision function is obtained as

(7) $$f\left(d\right)=\mathrm{sgn}\left(\sum_{i=1}^{n}\alpha_{i}l_{i}\left(d_{i}\cdot d\right)+b\right).$$

In this paper, the *n*-th order polynomial kernel ($\chi \left(d_{i},d_{j}\right)={\left(d_{i}^{T}d_{j}+c\right)}^{n},c>0$) and radial basis function (RBF) kernel ($\chi \left(d_{i},d_{j}\right)=exp\left(-{∥d_{i}-d_{j}∥}^{2}/\left(2\sigma \right)\right),\;\sigma \neq 0$) is applied to handle the non-linearly separated dataset. Considering that the training dataset is mapped into high dimensional space, non-linear transformation of the input vector $\varphi \left(d\right):R^{2}\to F$ should be applied, by which an optimal hyperplane can be obtained in the mapped high-dimensional feature space *F*. With the kernel functions in which $\chi \left(d_{i},d_{j}\right)=\varphi \left(d_{i}\right)\cdot \varphi \left(d_{j}\right)$ the decision function of SVM after the nonlinear transformation is φ(d) written as [18]

(8) $$f\left(d\right)=\mathrm{sgn}\left(\sum_{i=1}^{n}\alpha_{i}l_{i}\chi \left(d_{i}\cdot d_{j}\right)+b\right).$$

### 3.2. Naïve Bayes Classifier

The naïve Bayes classifier is based on Bayes’ theorem with the independence assumption that all features are uncorrelated. The Bayes’ theorem is expressed as follows:

(9) $$P\left(l\;|\;d\right)=\frac{P\left(d\;|\;l\right)\cdot P\left(l\right)}{P\left(d\right)},$$

where P(l|d) denotes likelihood, P(d|l) is the posterior probability, and P(d) and P(l) represent the evidence and predictor prior probability, respectively [19]. Equation (9) can be rewritten as Equation (10) using the chain rule and conditional independence assumptions,

(10) $$P\left(l_{j}\;|\;d\right)=\frac{\prod_{i=1}^{n}P\left(d_{i}\;|\;l_{j}\right)\cdot P\left(l_{j}\right)}{P\left(d\right)}.$$

By applying the maximum posterior rule, the probability can be obtained by maximizing only the numerator equation. In binary classification using the NB model, $l_{j}$ is considered to be +1 or −1. The data label is determined as follows,

(11) $$\hat{l}=\arg\max\prod_{i=1}^{n}P\left(d_{i}\;|\;l_{j}\right)P\left(l_{j}\right),$$

under the maximum posterior rule. $\hat{l}$ denotes the maximum a posteriori class that is determined by maximizing the classification performance [20].

### 3.3. Logistic Regression

As a regression method, LR calculates the probability that the realization of the output variable falls into a proper category. LR considers the conditional mean to obtain a probability value. The regression model $\beta^{T}d=\beta_{0}+\beta_{1}d_{1}+\beta_{2}d_{2}+\cdots +\beta_{n}d_{n}$, where β is a parameter vector, is to be established. Following the regression model and log-odds, the specific equation of the logistic function is as follows,

(12) $$\theta \left(\beta ,d\right)=\frac{1}{1+e^{-\beta^{T}d}}=y,$$

where *y* is the probability that the label indicates +1 in binary dataset. *y* considers a rational value between 0 and 1. To estimate the optimal parameter β that minimizes the regression model error caused by the noise components in measured dataset, the objective function is derived with the log-likelihood method that is the logarithm of the maximum likelihood method [21]. The objective function based on the log-likelihood estimation of L(β) is expressed as follows:

(13) $$\begin{array}{cc} \ln\;L(\beta ) & =\sum_{i=1}^{n}\left[y_{i}\ln\left(\theta \left(\beta ,d_{i}\right)\right)+\left(1-y_{i}\right)\ln\left(1-\theta \left(\beta ,d_{i}\right)\right)\right]\\ \; & =\sum_{i=1}^{n}\left[y_{i}\left(\beta^{T}d_{i}\right)\right]-\sum_{i=1}^{n}\left[\ln\left(1+e^{\beta^{T}d_{i}}\right)\right]. \end{array}$$

Based on Equation (13), the objective function is derived to find the optimal parameter β that induces Equation (13) to have the largest value. To determine β, several optimization algorithms, e.g., the gradient descent, Broyden–Fletcher–Goldfarb–Shanno algorithm (BFGS), limited-memory BFGS, quasi-Newton, and Newton–Raphson method were applied [22,23,24]. Despite using the complete optimization method and general dataset, the classification performance with new data is often below expectations. Moreover, overfitting can occur in the case of high-dimensional regression models.

Many studies have been conducted to improve the classification performance and overcome the weakness of the LR algorithm caused by a small training set and low-feature dataset. To reduce misclassification, model selection algorithms, such as the Akaike information criterion and Bayes information criterion, are combined with the LR model [25]. However, earthquakes and artificial explosions occur irregularly, and are difficult to obtain a large number of dataset for training and selection.

In this paper, teh RLR method is used to discriminate between earthquakes and artificial explosions and to solve the overfitting problem of the regression model. Moreover, the regularized term of the RLR method improves the classification performance to overcome the small dataset problem that is relatively susceptible to noise components in a dataset compared with a large dataset.

By inserting the regularized term into the objective function in Equation (13), the objective function becomes penalized. With the regularized term, the objective function J(β) with constant *p* induces appropriate results by adjusting the optimal parameter λ to control the trade-off between the training data adjustment and misclassification avoidance caused by overfitting and underfitting. As represented in Equation (14), the RLR is designed with $L_{2}$-regularization that is known as a ridge-regularized term.

(14) $$J\left(\beta \right)=-\sum_{i=1}^{n}\left(y_{i}\left(\beta^{T}d_{i}\right)\right)+\sum_{j=1}^{n}\left(\ln\left(1+e^{\beta^{T}d_{j}}\right)\right)-\lambda \sum_{k=0}^{p}\left(\beta_{k}^{2}\right).$$

### 3.4. ADASYN

Earthquake events occur more frequently than artificial explosion events. Differences in the incidence rate result in the imbalanced data collected at seismic stations. Despite the mathematical evidence of machine-learning algorithms, machine-learning classifiers exhibit limited discrimination performance based on an imbalanced dataset. Moreover, data imbalance issues to overcome are studied in seismology and various fields, such as medical science, computer vision, and economics [26,27,28,29].

In this paper, the ADASYN method is adopted for machine-learning algorithms to overcome classification error caused by data imbalance. The ADASYN algorithm is an oversampling method to balance between majority class and minority class that represent earthquakes and artificial explosions, respectively [30]. ADASYN algorithm generates synthetic data according to the imbalance ratio between the majority class and minority class. The generated synthetic data belong to the minority class to balance the majority class data [31].

In this paper, we implemented the ADASYN algorithm for four machine-learning methods. Algorithm 1 describes how to generate the synthetic dataset to balance between majority class and minority class using ADASYN pseudocode. The input of the suggested algorithm is a training dataset $\left(x_{i},y_{i}\right)$ that represents the feature vector and label, respectively. In the first step, the variable of *S*, that is the amount of synthetic dataset, is calculated based on the difference in data between the majority class ($A_{maj}$) and minority class ($A_{min}$) data. ν is used to determine the balance of the data generation required with ν∈[0,1].

Steps 2–4 are processed to obtain the number of synthetic data ($g_{i}$) induced by the product of β∈[0,1] and normalized ratio ($\bar{\rho_{i}}$), that is calculated according to the *K*-nearest examples ($\gamma_{i}$) in the minority class. To generate the synthetic dataset, the loop from step 5 to 8 is performed. The function randomlySelect(·) is used to randomly select $x_{zi}$, that is *K*-nearest neighbor of $x_{i}$. The output $u_{i}$ of Algorithm 1 is the generated data for the minority class.
**Algorithm 1** ADASYN**Input:** Imbalanced training data $\left(x_{i},y_{i}\right)$**Output:** Synthetic data in minority class $\left(u_{i}\right)$1:$S=\left(A_{maj}-A_{min}\right)\cdot v$2:$\rho_{i}=\gamma_{i}/K$3:$\bar{\rho_{i}}=\rho_{i}/\sum \gamma_{i}$4:$g_{i}=\beta \cdot \bar{\rho_{i}}$5:**for**$i<g_{i}$**do**6:    $x_{zi}=randomlySelect\left(x_{i}\right)$7:    $u_{i}=x_{i}+rand\left(0,1\right)\cdot \left(x_{zi}-x_{i}\right)$8:**end for**9:**return**$u_{i}$

## 4. Classification Performance Evaluation

The simulation comprises three different procedures to discriminate between earthquakes and artificial explosions and compares the performance of each machine-learning method. As the first step, an imbalanced seismic dataset was obtained from the United States Geological Survey (USGS) and the Incorporated Research Institutions for Seismology (IRIS), which recorded from the station on the Pacific Northwest Seismic Network (PNSN) during 2017 and 2020 [32].

The feature vectors were derived using the $A_{r}$ and $S_{r}$ methods, respectively. The $A_{r}$ method was applied to an imbalanced training dataset to configure the feature vector. As a preprocessing step before the implementation of the $S_{r}$ method, the time domain was converted into the frequency domain using FFT, and optimized frequency bands were selected from the spectrogram obtained by STFT. As a second step, to overcome data imbalances, we applied the ADASYN algorithm to maintain the balance of the data between the majority class and minority class by generating synthetic data for the minority class.

Machine-learning-based classifiers were applied using the balanced training dataset. Four classifiers were trained using a supervised model: SVM with RBF kernel function, NB, LR, and RLR. As a third step, to discriminate the seismic data, each classifier was applied to the new seismic dataset that was driven by the $A_{r}$ and $S_{r}$ methods.

**Figure 3:**
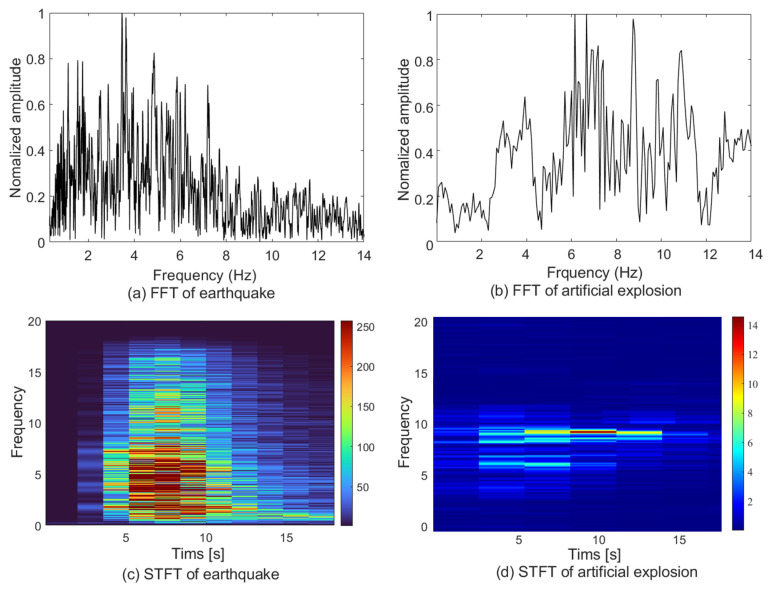
The FFT and STFT results for the seismic signals in Figure 1.

Figure 3 shows the results of STFT and FFT of the earthquakes and artificial explosions, respectively. To simulate seismic discrimination, an imbalanced seismic dataset in which the ratio of earthquake to artificial explosion is 7 to 3 was used to train the classifiers. The training dataset was obtained from seismic events, which exhibited a magnitude range of 1.8–3 and a local distance of 50–200 km. Earthquake and artificial explosion seismic data were measured at the same station under identical conditions of geological characteristics and measurement environment.

$A_{r}$ and $S_{r}$ method with the spectral frequency bands $f_{l1}$, $f_{l2}$, $f_{h1}$ and $f_{h2}$ as 1, 5, 6, and 10 Hz was adopted, respectively. Using the ADASYN method, the training dataset becomes a balanced dataset in which the ratio of earthquake to artificial explosion is 5 to 5. Various classifiers were built according to the same dataset by selecting different machine-learning methods. The RBF kernel function used in the SVM classifier has the parameter of σ=0.3. In addition, the fourth order polynomial kernel was used to configure the SVM classifier. The quasi-Newton method was used to optimize the objective functions of LR and RLR.

**Figure 4:**
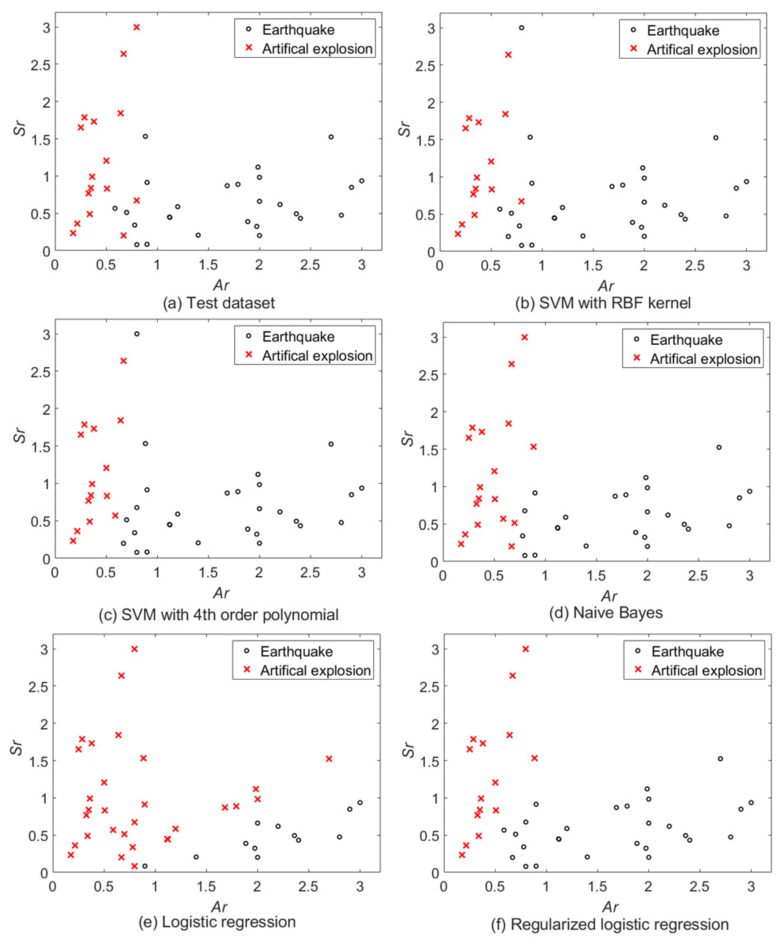
Test dataset and classification results of the ADAYSN-based machine-learning methods.

The value of λ=0.01 was selected as the optimal parameter of the RLR to avoid overfitting. Figure 4a shows the test dataset to verify the performance of the ADASYN-based classifiers. Figure 4b–f represent the classification results of the SVM classifier with RBF kernel with σ=0.3, SVM classifier with fourth order polynomial kernel, NB classifier, LR classifier, and RLR classifier with λ=0.01, respectively. Table 1 shows the numerical parameters used for the realization of seismic event classification models.

To compare the classification performances of SVM, NB, LR, and RLR, nine performance indicators (the ROC curve, AUC, sensitivity, specificity, F1-score, accuracy, MCC, YI, and FMI) were computed. The discrimination results are represented as +1 and −1. The result of +1 indicates that the test data are determined as an earthquake by the classifier. In contrast, −1 shows that the test data are identified as artificial explosion using the classifier. The classification outcomes have four possible results using actual labels and prediction results. The true positive (TP) and false negative (FN) indicate that the classifier accurately predicted the test data. On the contrary, the false positive (FP) and true negative (TN) imply that the classifier incorrectly predicted the test data. The confusion matrix for the binary classification is shown in Table 2.

The ROC curve is an effective measure of discrimination performance and a graphical analysis tool that was initially proposed in the field of signal detection [33]. The AUC value indicates the space under the ROC curve. The AUC value is 1 when the classifier is perfectly performed, whereas 0 describes random distinguishing. Sensitivity and specificity are the ability of the classification to identify correctly the binary class. The sensitivity represents the conditional probability that is positive. On the contrary, the specificity means the conditional probability that indicates negative. The two methods are defined as follows:

(15) $$\begin{array}{cc} \mathrm{Sensitivity} & =\frac{TP}{TP+FN},\\ \mathrm{Specificity} & =\frac{TN}{TN+FP}. \end{array}$$

F1-score is used to show the balance between specificity and sensitivity. A higher F1-score indicates that the specificity and sensitivity of the classifier represent good performance. However, the F1-score is 0 when the specificity or sensitivity is zero, which indicates the fault classification result. Moreover, the MCC is used to evaluate the measurement of the binary classifier. The MCC value is always between −1 and +1, indicating a perfect negative correlation and a perfect positive correlation [34]. The accuracy is the proportion of the dataset that is correctly classified.

YI is used primarily to determine the cutoff point of the ROC curve. Moreover, YI is used to emphasize the classifier’s sensitivity and specificity performance and to obtain the information that the classifier was able to avoid misclassification [35]. The FMI evaluates a similarity performance of binary classification. The FMI is scored between 0 and 1, in which 0 and 1 indicate fault classification result and accurate classification result, respectively [36]. The equations of the F1-score, accuracy, MCC, YI, and FMI are shown as

(16) $$\begin{array}{cc} F1-\mathrm{score} & =\frac{2TP}{2TP+FP+FN},\\ \mathrm{Accuracy} & =\frac{TP+TN}{TP+TN+FP+FN},\\ \mathrm{MCC} & =\frac{TP\cdot TN-FP\cdot FN}{\sqrt{\left(TP+FP)\cdot (TP+FN)\cdot (TN+FP)\cdot (TN+FN\right)}},\\ \mathrm{YI} & =\frac{TP}{TP+FN}-\left(1-\frac{TN}{TN+FP}\right),\\ \mathrm{FMI} & =\sqrt{\frac{TP}{TP+FP}\cdot \frac{TP}{TP+FN}}. \end{array}$$

**Figure 5:**
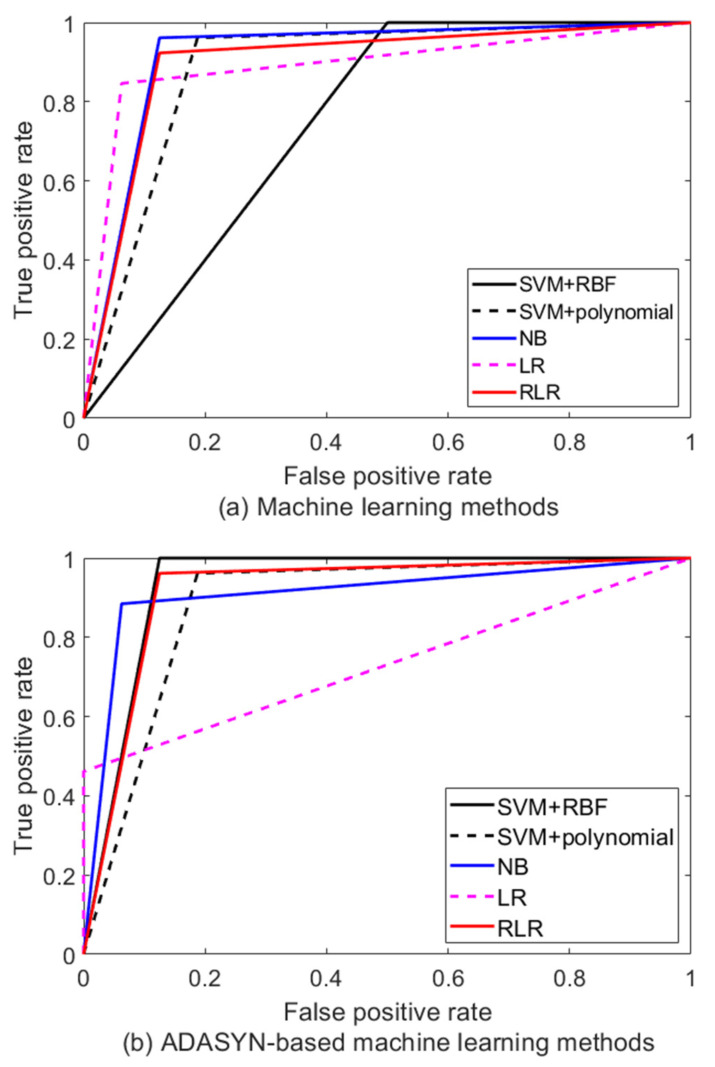
ROC curve of machine-learning methods and ADASYN-based machine-learning methods.

All performance metrics represent that closeness to the value of 1 indicates the best classification performance. The performance comparison for each ADASYN-based machine-learning method to discriminate between earthquakes and artificial explosions is listed in Table 3 and Table 4 and Figure 5. Table 3 and Figure 5a show the performance comparison results when ADAYSYN is not applied. As a proof of the datasets balancing performance, Table 4 and Figure 5b are the results after applying ADASYN to input datasets.

In Figure 5, the black solid, black dotted, blue solid, magenta dotted, and red solid lines denote the SVM with RBF kernel function, SVM with polynomial kernel, NB, LR, and RLR trained based on imbalanced and balanced data with the ADASYN algorithm, respectively. In Table 3 and Table 4, the fourth order polynomial kernel and RBF kernel with inverse variance (σ=0.3) were used for SVM realization. Based on the results in Table 3, the NB classifier demonstrated the best discrimination performance. The NB classifier trained using the imbalanced seismic dataset showed that it is better than other classifiers in six performance metrics as shown in Table 3.

However, this shows that the NB classifier performs worse than some other classifiers with respect to the balance between sensitivity and specificity. The NB classifier’s MCC and YI, which represent binary classification performance are evaluated as the same with RLR classifier’s MCC and YI. However, the NB classifier is not the only appropriate method for seismic classifier since SVM with RBF and LR exhibited the highest scores for sensitivity and specificity, respectively. Table 4 represents the performance of the ADASYN-based machine-learning methods.

The best performance of discrimination between earthquakes and artificial explosions was identified as an SVM classifier with RBF kernel (σ=0.3) based on the sensitivity, specificity, and AUC results that exhibit the classification rate. In addition, MCC, YI, and FMI of SVM with RBF kernel function score that are the performance indexes to verify the binary classification ability show the highest performance compared with other classifiers. Based on the results of comparing the discrimination ability and performance measurement score of classifiers with the imbalanced seismic dataset and the balanced seismic dataset derived by the ADASYN algorithm, the ADASYN-based SVM classifier with RBF kernel function (σ=0.3) showed outstanding performance.

## 5. Conclusions

In this paper, the main contribution of this research was the outstanding performance of seismic discrimination using machine learning in specific circumstances. The machine-learning models were trained by data of the time-frequency domain. In particular, the frequency domain data, $S_{r}$, was converted using the FFT method. Machine-learning methods were applied to discriminate between earthquakes and artificial explosions on the imbalanced dataset.

Machine-learning methods were proposed using feature vectors obtained from the $A_{r}$ method in the time domain and the $S_{r}$ method in the frequency domain. To convert to the frequency domain for the $S_{r}$ method, the FFT method was used. Moreover, to induce the optimal $S_{r}$ method, the best frequency bands with low-frequency range (1–5 Hz) and high-frequency range (6–10 Hz) were derived using the STFT method. To overcome the performance degradation owing to the imbalanced seismic dataset, the ADASYN algorithm was used to keep a balance between datasets of earthquakes and artificial explosions. Based on the balanced dataset, the SVM, NB, LR, and RLR models were designed.

Using the four ADASYN-based machine-learning methods, the discrimination of the new seismic dataset that comprised earthquakes and artificial explosions was executed. In order to confirm and compare the discrimination performance of the four machine-learning models, the fitness evaluation was verified using various performance metrics of ROC, AUC, sensitivity, specificity, F1-score, accuracy, MCC, YI, and FMI, respectively. Using the various performance indexes, SVM with RBF (σ = 0.3) was proven to be the best classifier for seismic event discrimination.

Through comparisons of each model, we proved which machine-learning method had an edge on seismic discrimination. The improved performance of seismic discrimination was able to reduce a number of false alerts about whether a seismic event is from nature or human activity regardless of small sample rates. Therefore, the discrimination ability can create a quantitative discrimination criterion, and an immediate response can be used for the realization of an early warning system.

## Figures and Tables

**Table 1:** Numerical parameters for the classification models.

Parameter	Method	Value
λ	RLR	0.01
*K*	ADASYN	5
σ	SVM with RBF	0.3
$f_{l1}$	$S_{r}$	1
$f_{l2}$	$S_{r}$	5
$f_{h1}$	$S_{r}$	6
$f_{h2}$	$S_{r}$	10
$\alpha_{i}$	SVM	0.7

**Table 2:** The confusion matrix for binary classification.

Confusion Matrix		Actual Class	
		Positive	Negative
Hypothesized class	Positive	TP	FP
	Negative	FN	TN

**Table 3:** Performance comparison of SVM with different kernel functions, NB, LR, and RLR trained based on an imbalanced seismic dataset.

	SVM		NB	LR	RLR
	Polynomial	RBF			
**Sensitivity**	0.9615	1.0000	0.9615	0.8462	0.9231
Specificity	0.8125	0.5000	0.8750	0.9375	0.8750
AUC	0.8870	0.7500	0.9183	0.8918	0.8990
**F1-score**	0.9259	0.8667	0.9434	0.8980	0.9231
Accuracy	0.9048	0.8095	0.9286	0.8810	0.9048
MCC	0.7974	0.6183	0.8478	0.7646	0.7981
YI	0.7740	0.5000	0.8365	0.7837	0.7891
FMI	0.9266	0.8745	0.9336	0.8896	0.9231

**Table 4:** Performance comparison of ADASYN-based SVM with different kernel functions, NB, LR, and RLR.

	SVM		NB	LR	RLR
	Polynomial	RBF			
**Sensitivity**	0.9615	1.0000	0.8846	0.4615	0.9615
Specificity	0.8125	0.8750	0.9375	1.0000	0.8750
AUC	0.8870	0.9375	0.9111	0.7308	0.9183
**F1-score**	0.9259	0.9630	0.9200	0.6316	0.9434
Accuracy	0.9048	0.9524	0.9048	0.6667	0.9286
MCC	0.7974	0.9014	0.8067	0.4961	0.8478
YI	0.7740	0.8750	0.8221	0.4615	0.8365
FMI	0.8686	0.9354	0.8839	0.7184	0.9037

## Data Availability

Data sharing not applicable.
